# Metastatic Melanoma Presenting as a Gastric Mass

**DOI:** 10.7759/cureus.11874

**Published:** 2020-12-03

**Authors:** Kevin Groudan, Wendy Ma, Kirti Joshi

**Affiliations:** 1 Internal Medicine, Baystate Medical Center, Springfield, USA; 2 Internal Medicine, University of Massachusetts, Worcester, USA; 3 Internal Medicine, University of Massachusetts Medical School Baystate, Springfield, USA

**Keywords:** melanoma, gastric mass, endoscopy

## Abstract

Melanoma is the most deadly form of skin cancer. While the jejunum, ileum, colon, and rectum are common gastrointestinal sites of metastasis, metastatic melanoma to the stomach is rare and usually not discovered until late in the disease. We report a patient who presented with weight loss and hematemesis; on esophagogastroduodenoscopy, a gastric mass was found, and pathology was consistent with melanoma.

## Introduction

Melanoma is the fifth most common malignancy in men and seventh in women [1]. Although nearly every organ of the human body can be affected by metastatic melanoma, gastric melanoma is very rare and often not detected until autopsy [2]. We report a patient who presented with weight loss and hematemesis, profoundly anemic, and on esophagogastroduodenoscopy (EGD) was found to have a gastric mass confirmed to be melanoma by immunohistochemical staining.

## Case presentation

**Figure 1:**
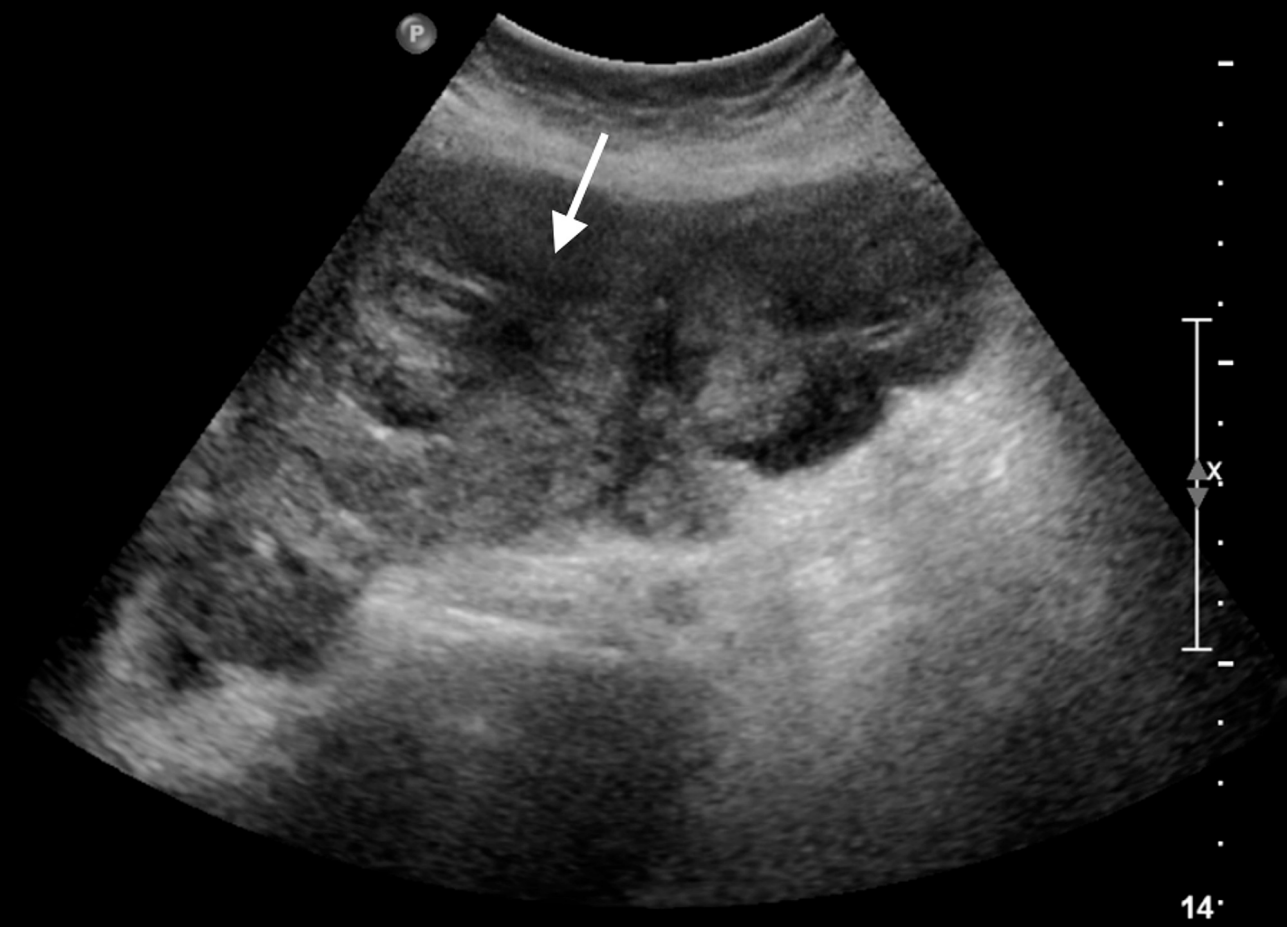
Liver ultrasound showing innumerable hepatic lesions concerning for malignancy

A 66-year-old Caucasian woman with no past medical history presented to our hospital with several weeks of worsening fatigue, shortness of breath on exertion, nausea, and three episodes of hematemesis. She also reported a 30-pound weight loss over the last three months. She had not seen a doctor in over 20 years. On arrival, she was tachycardic with a heart rate of 104 bpm. The physical exam was unremarkable. Admission labs were significant for a hemoglobin of 6.5 gm/dL (11.7-15.5 gm/dL), mean corpuscular volume of 78.3 femtoliters (80.0-100.0 femtoliters), leukocytosis of 15 k/mm3 (4.0-11.0 k/mm3), platelet count of 460 k/mm3 (150-460 k/mm3), aspartate aminotransferase of 127 gm/dL (0-32 gm/dL), total bilirubin of 2.7 mg/dL (0-1.2 mg/dL), alkaline phosphatase of 176 units/L (35-104 units/L), protime of 11.8 seconds (9.7-12.2 seconds), international normalized ratio 1.2 (0.9-1.1), and partial thromboplastin time 23.6 seconds (24.3-33.1 seconds) . There were no baseline labs. A liver ultrasound revealed innumerable hepatic lesions suspicious for metastasis (Figure 1).

**Figure 2:**
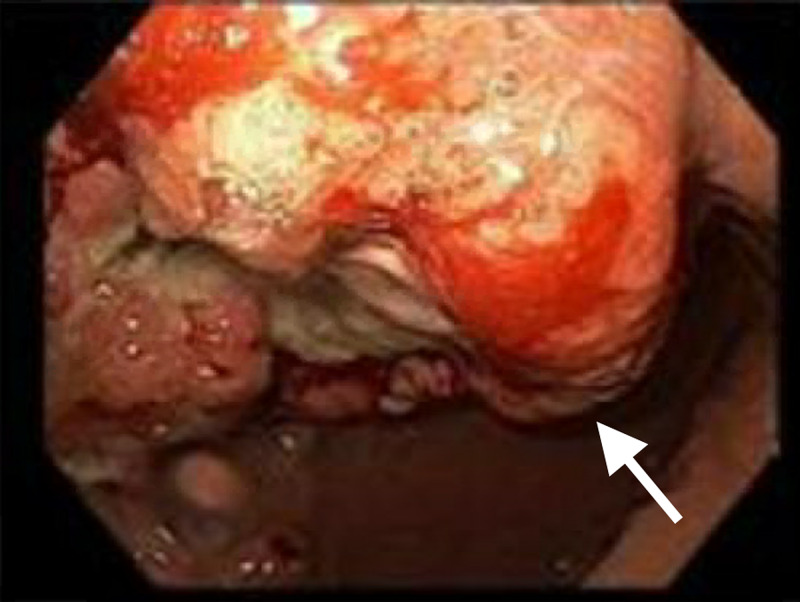
EGD showing an ulcerated and fungating mass in the gastric antrum, concerning for gastric malignancy

The patient was resuscitated with packed red blood cells and scheduled for EGD and colonoscopy for concern for gastrointestinal bleed and malignancy. EGD was significant for an ulcerated and fungating 3 cm mass in the gastric antrum, highly concerning for gastric malignancy (Figure 2). 

**Figure 3:**
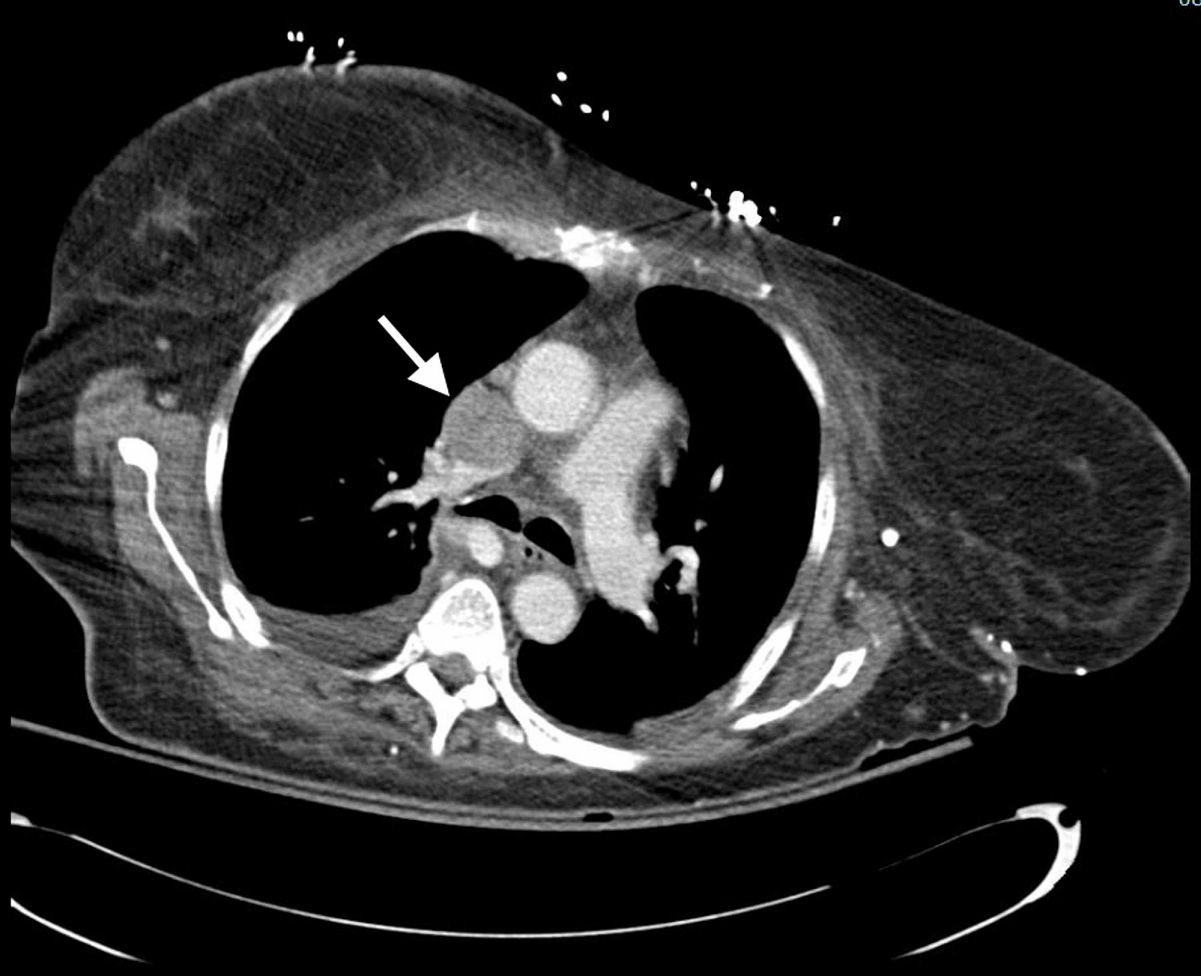
CT of chest showing a completely occlusive thrombus of the superior vena cava

Biopsies were obtained and sent for histopathology. Colonoscopy was unremarkable. A computed tomography (CT) of the abdomen, pelvis, and chest with contrast was significant for a completely occlusive thrombus of the superior vena cava (Figure 3), partially occlusive thrombi of the inferior vena cava and left subclavian vein, numerous large liver metastases, bilateral large adrenal metastases, a bulky mass in the gastric antrum, and a probable thrombus of the right external iliac vein.

**Figure 4:**
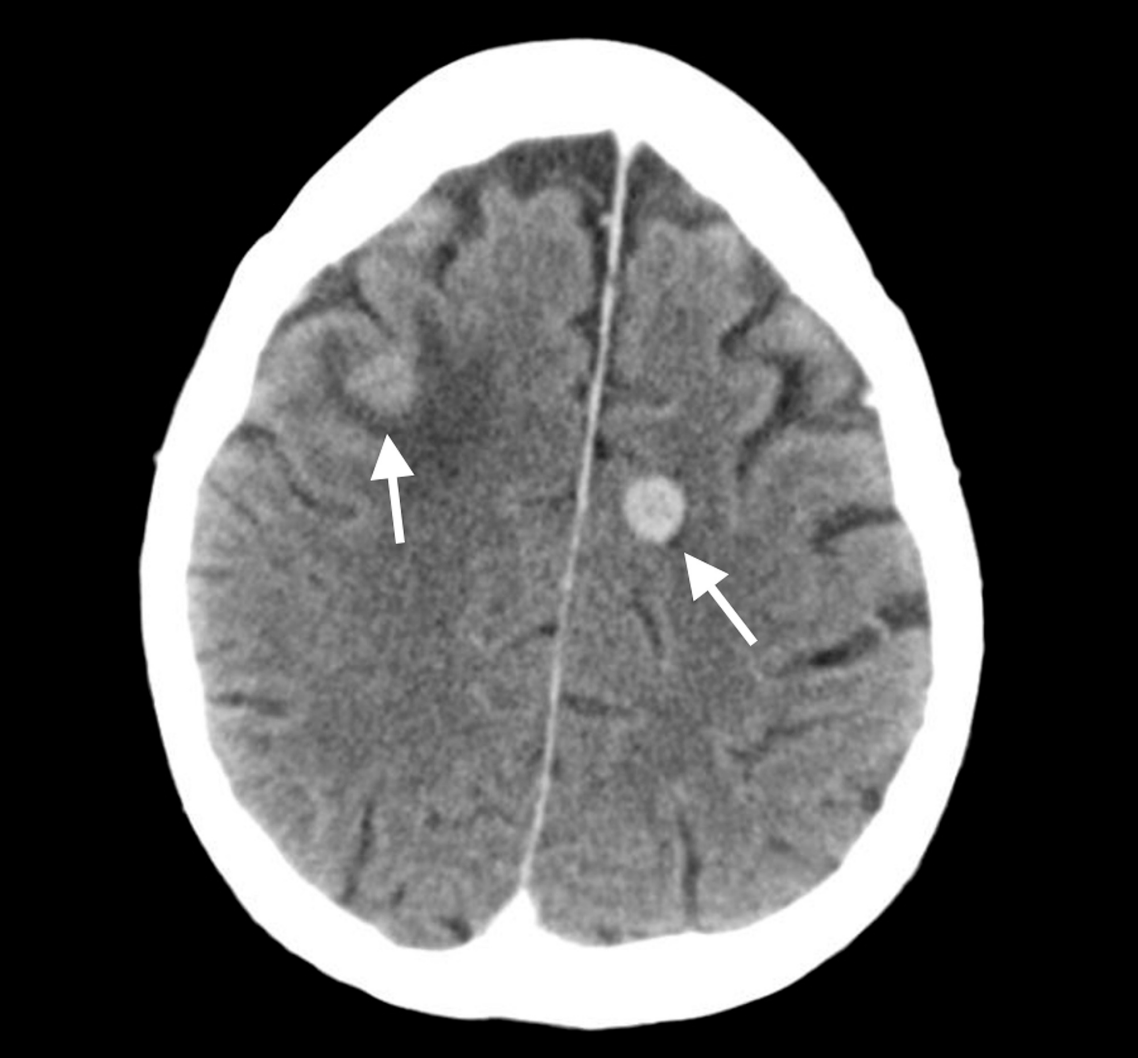
CT of the head showing multi-focal enhancing masses with surrounding edema

A CT of the head with contrast further showed multi-focal enhancing masses with surrounding edema (Figure 4).

**Figure 5:**
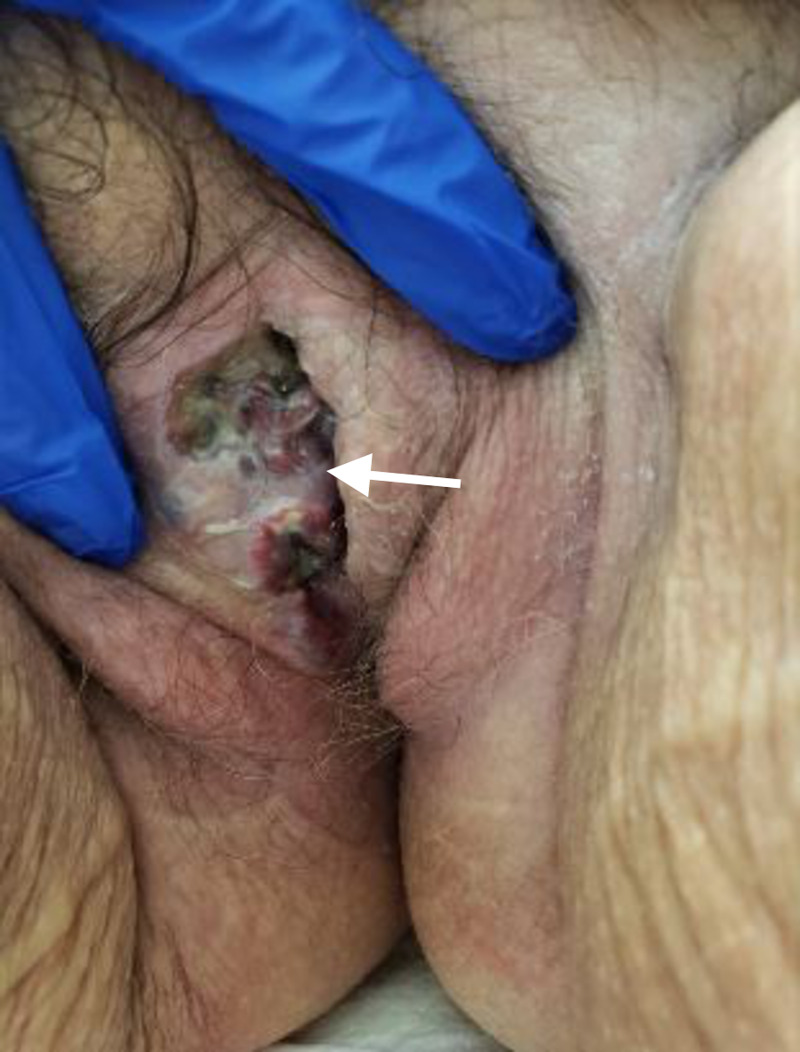
Skin exam showing a melanoma appearing vulvar lesion

Immunohistochemical staining of the biopsy specimen returned positive for Melan-A and c-Kit, consistent with a diagnosis of melanoma. On subsequent skin examination, a melanoma appearing vulvar lesion was noted (Figure 5).

The patient underwent palliative radiation to her stomach to stop tumor bleeding and was scheduled for outpatient immunotherapy. She was discharged on enoxaparin for her extensive thromboses however cautioned to watch for signs of gastrointestinal bleeding in the setting of her gastric metastasis. 

## Discussion

Melanoma is the most lethal skin cancer. Despite accounting for 5% of cutaneous malignancies, melanoma makes up the majority of skin cancer-related deaths [3]. Malignant melanoma most commonly metastasizes to the skin, lung, brain, liver, bone, and intestine [4]. Although malignant melanoma of the gastrointestinal tract is common, gastric localization is unusual and not well reported [5]. Symptoms of gastric melanoma include epigastric discomfort, nausea, vomiting, weight loss, hematemesis, and melena [6]. Our patient presented with nausea, weight loss, and hematemesis. Due to their rarity, gastric melanoma metastatic disease is often confused for other cancers of the stomach, including primary gastric carcinomas and lymphomas and metastasis from breast and lung carcinomas. Other conditions that could mimic gastric melanoma metastatic disease include peptic ulcer disease, gastritis, bowel obstruction, and gastroenteritis [7].

As with other gastrointestinal malignancies, iron deficiency anemia is commonly associated with cutaneous melanomas metastatic to the stomach [8]. While they are often first identified by radiologic studies, EGD is necessary for definitive diagnosis by direct visualization and biopsy of the tumor. The endoscopic appearance of gastric melanomas varies [9]. One case series included reports of gastric metastasis from cutaneous melanoma presenting as polypoid masses, nodules, or ulcers, and in one instance, a dark, flat macule. One presentation in five patients was described as an umbilicated sub-mucosal melanotic or regular stained nodule with ulceration on top. Histologically, gastric melanomas, when stained with hematoxylin and eosin, reveal large nests of epithelioid pigmented or non-pigmented cells. Gastric melanoma tumor markers include S100, HMB45, and MART1; these markers are seen in most cutaneous melanomas and are not unique to metastatic melanoma to the stomach [10].

There are no guidelines for the treatment of primary gastric melanomas. Treatment for metastatic melanomas irrespective of the site of metastasis is similar. As the stomach is rarely the only site of metastasis, systemic therapy is generally used. Surgery is generally not performed unless the patient is a surgical candidate and has complications that could be relieved with surgery [10]. Given our patient’s poor condition and extensive distant metastases to brain, kidney, liver, and major vasculature, she was not a surgical candidate. There are cases of gastric melanoma managed with radiation therapy, usually after surgical resection [2]. However, treatment by radiation is not well reported as melanoma is generally a radio-resistant tumor [11]. In our patient, radiation was used palliatively to stop tumor bleeding.

Most cutaneous melanoma cases diagnosed by a gastric mass have a poor prognosis from a delay in diagnosis. The average time for a primary cutaneous melanoma to metastasize to the gastrointestinal tract is estimated to be 52 months [2]. Additionally, gastric mucosa has a rich lymphatic and vascular supply, making gastric metastases particularly aggressive. Median survival is usually from four to six months [12, 13].

## Conclusions

In conclusion, we present a rare case of metastatic melanoma diagnosed by biopsy of a gastric mass. Our case adds to the literature that the stomach can be a metastatic site for melanoma and should be kept as a differential in the workup of gastric mass.
